# Stem Cell Factor SALL4 Represses the Transcriptions of PTEN and SALL1 through an Epigenetic Repressor Complex

**DOI:** 10.1371/journal.pone.0005577

**Published:** 2009-05-18

**Authors:** Jiayun Lu, Hawon Jeong, Nikki Kong, Youyang Yang, John Carroll, Hongbo R. Luo, Leslie E. Silberstein, YupoMa, Li Chai

**Affiliations:** 1 Department of Pathology, Joint Program in Transfusion Medicine, Brigham and Women's Hospital/Children's Hospital Boston, Harvard Medical School, Boston, Massachusetts, United States of America; 2 Division of Laboratory Medicine, Nevada Cancer Institute, Las Vegas, Nevada, United States of America; Istituto Dermopatico dell'Immacolata, Italy

## Abstract

**Background:**

The embryonic stem cell (ESC) factor, SALL4, plays an essential role in both development and leukemogenesis. It is a unique gene that is involved in self-renewal in ESC and leukemic stem cell (LSC).

**Methodology/Principal Findings:**

To understand the mechanism(s) of SALL4 function(s), we sought to identify SALL4-associated proteins by tandem mass spectrometry. Components of a transcription repressor Mi-2/Nucleosome Remodeling and Deacetylase (NuRD) complex were found in the SALL4-immunocomplexes with histone deacetylase (HDAC) activity in ESCs with endogenous SALL4 expression and 293T cells overexpressing SALL4. The SALL4-mediated transcriptional regulation was tested on two potential target genes: PTEN and SALL1. Both genes were confirmed as SALL4 downstream targets by chromatin-immunoprecipitation, and their expression levels, when tested by quantitative reverse transcription polymerase chain reaction (qRT-PCR), were decreased in 293T cells overexpressing SALL4. Moreover, SALL4 binding sites at the promoter regions of PTEN and SALL1 were co-occupied by NuRD components, suggesting that SALL4 represses the transcriptions of PTEN and SALL1 through its interactions with the Mi-2/NuRD complex. The *in vivo* repressive effect(s) of SALL4 were evaluated in SALL4 transgenic mice, where decreased expressions of PTEN and SALL1 were associated with myeloid leukemia and cystic kidneys, respectively.

**Conclusions/Significance:**

In summary, we are the first to demonstrate that stem cell protein SALL4 represses its target genes, PTEN and SALL1, through the epigenetic repressor Mi-2/NuRD complex. Our novel finding provides insight into the mechanism(s) of SALL4 functions in kidney development and leukemogenesis.

## Introduction

The human SAL gene family, SALL1, SALL2, SALL3 and SALL4, was originally cloned on the basis of DNA sequence homology to the Drosophila homeotic gene spalt [1]–[3]. In Drosophila, SAL is a zinc finger transcription factor and required for development of the posterior head and anterior tail segment in early embryos, as well as for later stages of organogenesis and adult wing morphogenesis [4], [5]. SAL-related genes have been isolated from Caenorhabditis elegans [6], [7], fish [8], [9], Xenopus [10], [11], mice [12], and humans [1], [13]. Each of these homologues is expressed during embryonic development, as well as in specific adult tissues.

Human SALL4 mutations are associated with the Duane-radial ray syndrome (DRRS, OMIM#126800, also known as Okihiro syndrome), which is a human autosomal-dominant syndrome involving multiple organ defects, including kidney malformation [1], [13]–[15]. SALL4 is also essential for maintenance of pluripotent and self-renewal properties of embryonic stem cells (ESCs) by interacting with other two key regulators in ESCs, NANOG and OCT4 [16]–[19]. Loss of SALL4 expression in mouse ESCs results in downregulation of OCT4 and spontaneous differentiation. Significant zygotic SALL4 transcription in mice occurs after the 4-cell stage and continues to increase until the blastocyst stage. By 10.5 days postcoitum, SALL4 is detectable mainly in the stem/progenitor populations in various organ systems including the brain and bone marrow of the embryo and later in the adult. This may suggest that SALL4 is not only involved in ESCs but also in adult stem cells [19]. Moreover, we have reported that SALL4 is constitutively expressed in human leukemia cell lines and primary acute myeloid leukemia (AML) cells. We have generated a SALL4 transgenic mouse model in which SALL4B, one of the SALL4 isoforms, is overexpressed. These mice exhibit a pre-leukemic dysplastic phase and subsequently develop AML transformation that is transplantable [20], [21]. In addition, 50% of these transgenic mice developed cystic kidneys. Together, these characteristics indicate that SALL4 is a critical gene for ESC properties, kidney development, and leukemogenesis, and is involved in the biological functions of various types of stem cells. Defining the precise mechanism(s) and downstream target genes of SALL4 is of great importance to the field of stem cell study.

Recent studies have demonstrated that a conserved N-terminal 12 amino acid domain of SALL1 and FRIEND OF GATA1 (FOG-1) is sufficient for recruiting Mi-2/Nucleosome Remodeling and Deacetylase (NuRD) complex and mediating transcriptional repression [22]–[24]. It is known that acetylation status of histones is associated with the regulation of gene transcription. Generally, when histones are acetylated, chromatin becomes loosely packed euchromatin and gene expression is activated. In contrast, deacetylated histones lead to condensed heterochromatin and repressed gene expression [25]. In mammalian cells, there are two major histone deacetylation complexes, Mi-2/NuRD and Sin3. These two protein complexes share four common components, HDAC1, HDAC2, RbAp46 and RbAp48. Mi-2/NuRD complex additionally includes Mi-2, MTA-1, MTA-2, p66 and MBD3. Among these eight factors, HDAC1 and HDAC2 are histone deacetylases, RbAp46 and RbAp48 are histone binding proteins, and Mi-2 is a chromatin remodeling ATPase. Therefore, histone deacetylation and chromation remodeling ATPase activities are uniquely linked in this single protein complex Mi-2/NuRD, whose function is to produce compactly packed, hypoacetylated nucleosomes that switch an active, hyperacetylate promoter to its inactivated state [26].

In this study, we report that SALL4, a protein containing the same conserved 12 amino acid motif [22], [24], [27], associates with the NuRD complex and functions as a strong transcription repressor. Moreover, we tested the effect of SALL4-NuRD complex in two SALL4 target genes: Phosphatase and Tensin Homolog (PTEN) and SALL1. These two genes are chosen for our studies based on their roles(s) in ESC, leukemogenesis and kidney development, which are closely relevant to the biological functions of SALL4.

PTEN is a tumor suppressor gene and essential for both normal hematopoiesis and leukemogenesis [28]–[30]. It has been shown that deletion of PTEN results in generation of leukemic stem cells as well as depletion of normal hematopoietic stem cells [28], [31]–[32]. Absence of PTEN can also alter ESC properties and impair kidney development [33]–[35]. SALL1 is a stem cell factor in kidney development [36], and is mutated in patients with Townes-Brocks syndrome (TBS), whose features include renal malformations [37]–[39]. Genetic targeting of mouse SALL1 results in severe renal dysplasia or complete agenesis, indicating that SALL1 plays an essential role in early kidney development. In addition, SALL1 is expressed in both human and mouse ESCs, and has been associated with a potential role in leukemogenesis [40].

In this study, we have demonstrated that SALL4 represses the expression of PTEN and SALL1 by interacting with the NuRD complex. This inhibition may at least contribute in part to the SALL4-induced leukemia and kidney abnormalities in the transgenic mice.

## Materials and Methods

### Mice

Generation of transgenic SALL4B mice has been previously described [20].

### Immunocytochemistry

293T cells were transiently transfected with SALL4A or SALL4B constructs. After 48 h, the transfected cells were fixed with 4% paraformaldehye-PBS for 20 min at room temperature, then permeabilized in 0.5% Trixon X-100 in PBS, and then blocked with 3% BSA in PBS for 1 h at room temperature. Blocked cells were incubated with anti-SALL4 antibody for overnight at 4°C followed by incubation with secondary antibody (Alexa Flour 488 conjugated anti-rabbit IgG, Molecular Probes, Invitrogen, Carlsbad, California) diluted at 1∶1000 in PBS for 30 min at room temperature.

### Reporter Assays

Full length SALL4B was cloned in frame to the GAL4 DNA binding domain. As reporter constructs, pGAL4-tk-Luc was used, which contains the luciferase gene under the control of the thymidine kinase (tk) promoter with upstream GAL4 binding sites. The 293T cells were transiently transfected with the GAL4-SALL4B fusion plasmid, luciferase reporter plasmid (GAL4-tk-Luc), and cytomegalovirus-b-galactosidase control plasmid. After 48 h, the transfected cells were extracted with lysis buffer and used for luminescence measurements with luminometer. These experiments were performed in triplicate.

### Cell culture

All cell cultures were maintained at 37°C with 5% CO_2_. 293T cells were cultured in Dulbecco's modified Eagle's medium (DMEM, Invitrogen) supplemented with 10% fetal bovine serum (FBS, Invitrogen) and 50 U/ml penicillin/streptomycin (Invitrogen). D3 mouse ES cells (CRL-1934, ATCC) were cultured on 0.1% gelatin-coated plates in DMEM supplemented with 15% FBS, 2 mM L-Glutamine, 1× nucleoside mixture, 0.1 mM β-mercaptoethanol, and leukemia inhibitory factor (LIF, 1000 units/ml: ESGRO, Chemicon International).

### Purification of SALL4 protein complex (Mass Spectrometry)

293T cells expressing FLAG-tagged SALL4B or an empty vector were grown to confluence on culture dishes in DMEM containing 10% FBS. Cells were lysed with buffer A (50 mM Tris-HCl, pH 7.5, 100 mM NaCl, 2 mM MgCl2, 10% glycerol, 1% Nonidet P-40, 2 mM Na3VO4, 10 mM NaF, and protein inhibitor cocktail (Sigma). A total cell extract was prepared after centrifugation (14,000×g for 20 min) and then immunoprecipitated with anti-FLAG M2 agarose (Sigma) for 5 hr at 4°C. After washing with buffer A 10 times, bound proteins were eluted twice with 0.25 mg/ml FLAG peptide (Sigma), resolved on 4–15% or 7% SDS-PAGE (Bio-Rad), subjected to silver staining, and analyzed by mass spectrometry.

### HDAC activity assay

HDAC assays were performed using the HDAC fluorimetric assay kit (Upstate) according to the manufacturer's instructions. Briefly, lysates from 293T cells expressing SALL4 or from ES cells were immunoprecipitated with anti-FLAG antibody (for 293T–SALL4B transfectants), anti-SALL4 antibody (for 293T-SALL4A transfectants and ES cells) or rabbit IgG (as a control). The immunocomplexes were washed with lysis buffer 5 times and then incubated with fluorescene-producing substrate (Fluor de lys substrate) with or without Trichostatin A (TSA, 200 nM) for 40 min at room temperature with rocking. These assays were stopped by adding Fluore de Lys developer (50 ul). Fluorescent, deacetylated substrate was detected in a 96-well plate using Victor3V 1420 Multilabel Counter (Perkin Elmer).

### Co-Immunoprecipitation

293T cells were transfected with pcDNA3-SALL4A or SALL4B and harvested after 48 hours. Nuclear proteins of SALL4A or SALL4B-expressed 293T cells and D3 cells were prepared with Nuclear Extract kit (Active Motif) according to manufacturer's instructions. 1 mg of nuclear protein was incubated with 5 µg of anti-SALL4 antibody or rabbit IgG (Upstate) at 4°C overnight. 20 µl protein G beads (Sigma) were added into protein-antibody mixture and incubated for 3 hours at 4°C. Then the beads were washed 5 times with 1 ml of wash buffer containing 0.1% NP-40. Interacting proteins were eluted with 50 µl 1× protein sample loading buffer. The eluted sample was loaded onto 4%–15% SDS-PAGE gels (Bio-Rad) and the following Western blots were performed with antibodies against HDAC1 (H6287, Sigma), HDAC2 (H2663, Sigma), MTA-2 (sc-9402, Santa Cruz), Mi-2 (sc-11378, Santa Cruz) and Sin3A (sc-5299, Santa Cruz).

### ChIP (Chromatin immunoprecipitation)

293T cells were transiently transfected either with a full length SALL4A or SALL4B or with an empty vector. Cells were cross-linked, lysed, and sonicated. Chromatin immunoprecipitations were conducted with a polyclonal anti-SALL4, anti-HDAC2 (H2663, Sigma), or anti-acetylated histone H3 (Ac-H3, Upstate) antibody. With quantitative real-time PCR, PTEN and SALL1 primers specific to a 150-bp ∼250-bp region of the promoters were used to validate pulldown DNA fragments. Fold enrichment was calculated after normalization with input (no antibody added) of all three types of transfected cells.

### Quantitative real-time PCR (qPCR)

Pulldown DNA from ChIP was measured by UV spectrophotometry. About 60 ng of DNA was utilized to perform qRCR with iTaq SYBR Green Supermix (Bio-Rad). Reactions were carried out in duplicates.

### Quantitative real-time RT-PCR (qRT-PCR)

Total RNA was extracted with Trizol reagent (Invitrogen) according to manufacturer's instructions, and the concentration was measured by UV spectrophotometry. 100 ng of total RNA was applied to conduct qRT-PCR by using iScript One-Step RT-PCR Kit with SYBR Green (Bio-Rad). The average threshold cycle for each gene was determined from duplicate reactions and the expression level was normalized to glyceraldehyde 3-phosphate dehydrogenase (GAPDH).

### Mouse Magnetic resonance imaging (MRI) imaging

This group of experiments was done by the core facility of Small Animal MRI Laboratory at Brigham and Women Hospital according to a previously published procedure [41]. Briefly, mouse MRI measurements were performed using a 4.7 T Bruker Avance horizontal bore system equipped with a 200 mm inner diameter gradient set capable of 30 G/cm gradient strength or a 7T Bruker Pharmascan system. The mice were anesthetized with 1% isoflurane in an oxygen/air mixture. The animals' respiratory and cardiac rates were monitored using Biotrig Software (4.7T) or SA Instruments monitoring device (Stony Brook, NY for 7T system). The animals were imaged on the 4.7T system with RARE sequence (TR = 2000 ms, TE effect = 25 ms) in the coronal and axial planes with a 1 mm slice thickness and with the number of slices sufficient to cover entirety of the kidneys. A matrix size of 128×128 and field of view (FOV) of 2.5×2.5 cm^2^ were used for the images.

### Mouse Tissues and Hematoxylin and Eosin Staining

Methods on mouse tissue collection and hematoxylin and eosin (H&E)staining were previously described [20].

## Results

### SALL4 functions as a transcriptional repressor

**Figure 1:**
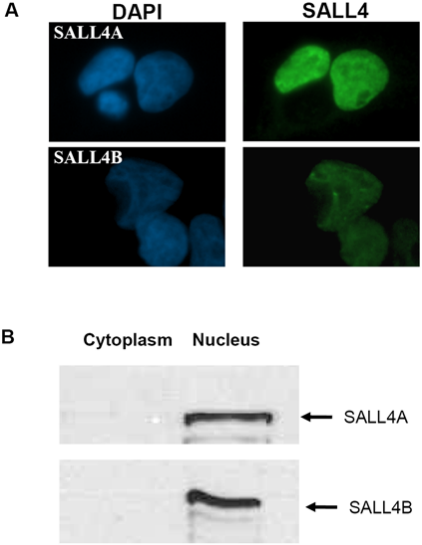
Nuclear localization of SALL4. (A) Immunofluorescence of 293T cells transfected with either SALL4A (upper panel) or SALL4B (lower panel). Cells were stained with polyclonal anti SALL4 antibody (right) and DAPI (left). Both SALL4A and SALL4B were observed only in the nuclei of transfected. (B) Cellular fractionation with Western blotting showing SALL4A and SALL4B in the nuclei. Lysates from SALL4A or SALL4B-expressed 293Tcells were fractionated. 50 ug of subcellular fractions were separated on the SDS-PAGE and probed with the anti-SALL4 antibody.

The presence of multiple characteristic zinc finger domains in SALL4 suggests that SALL4 could be a transcription factor. To verify this, we first examined the subcellular localization of two variants of SALL4 proteins (SALL4A and SALL4B) by immuno-fluorescent staining and cellular fractionation with Western blotting. As shown in Figure 1A, when SALL4A and SALL4B were overexpressed in 293T cells, both proteins were localized only to the nuclei of the cells. The resulting cytoplasmic and nuclear fractions were analyzed by Western blotting and SALL4A and SALL4B were again detected exclusively in the nuclei (Figure 1B).

**Figure 2:**
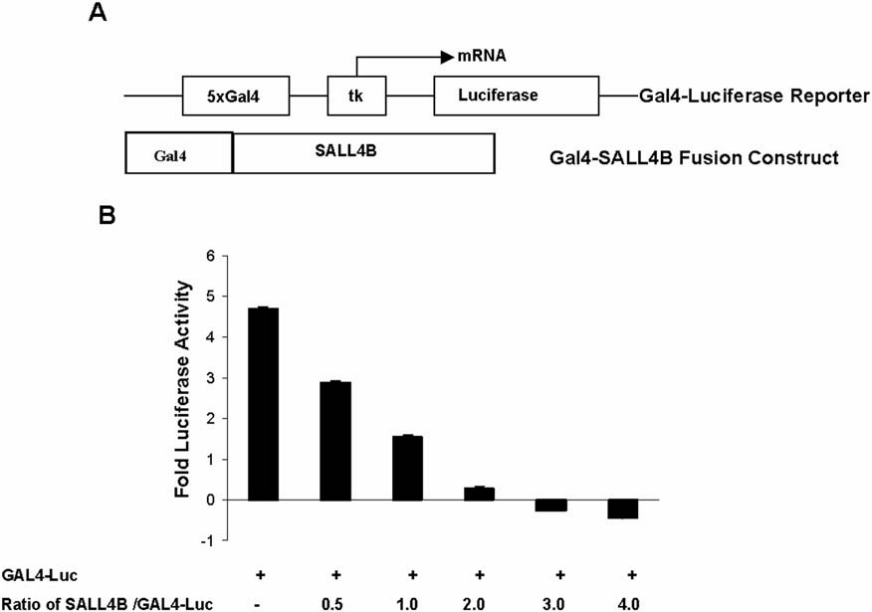
Transcriptional repression by tethered SALL4B. (A) Schematic representation of the GAL4 fusion protein (GAL4–SALL4B) and luciferase reporters (GAL4-tk-Luc). 5×Gal4, five copies of the GAL4-binding site; tk, thymidine kinase core promoter. (B) Constructs expressing SALL4B fused to the C-terminus of GAL4 (1–93) were transfected into 293T cells, along with the reporter GAL-tk-Luc. Luciferase activities were normalized to the internal ß-galactosidase control. Different molar ratios of GAL4-SALL4B and GAL4-tk-Luc were used for transfection.

Secondly, we fused the SALL4B isoform to the minimum DNA-binding domain of the yeast transcription factor GAL4 (1–93) and used the tk-GALpx3-Luc reporter construct to examine the effect of the SALL4B isoform on the activity of the reporter containing the GAL4 binding site. GAL4–SALL4B remarkably repressed transcription in a dose-dependent manner (Figure 2), which, combined with the nuclear localizations of SALL4 proteins, supports the notion that SALL4 functions as a strong transcriptional repressor. We chose the SALL4B isoform for this functional assay because we generated a transgenic SALL4B mouse model that can be used for *in vivo* functional study correlation.

### SALL4 associates with Mi-2/nucleosome remodeling and deacetylase (NuRD) complex *in vitro*

**Figure 3:**
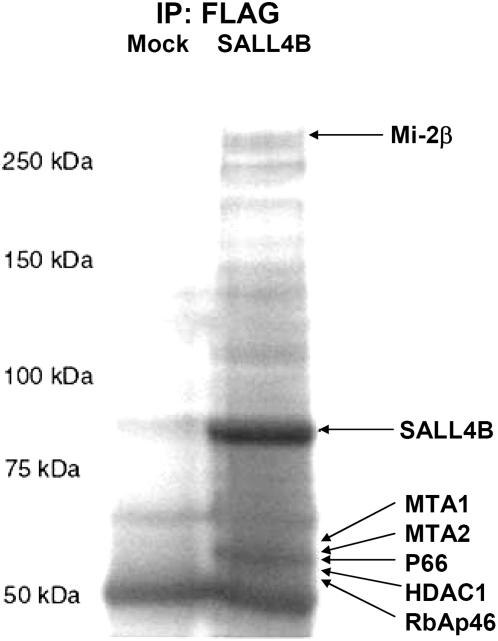
SALL4B-bound proteins include the components of Mi-2/NuRD complex. Extracts from 293Tcells expressing FLAG-SALL4B or mock vector only were immunoprecipitated with anti-FLAG M2 agarose. SALL4B-bound proteins were fractionated by SDS-PAGE, detected by silver staining and identified by tandem mass spectrometry.

In order to understand the mechanism(s) whereby SALL4 represses gene transcription, and to identify cofactors of SALL4, we transfected FLAG-tagged SALL4B vector into 293T cells and purified the SALL4B-containing protein complex. Several bands were observed in only FLAG-SALL4B bound proteins but not in mock controls (Figure 3). Followed by tandem mass spectrometry analysis, in the 200-kDa band 42 peptides were identified as Mi-2β which has been shown to be the specific component of Mi-2/NuRD complex. In addition, the other bands were identified as MTA1 (7 peptides), MTA2 (18 peptides), p66 (16 peptides), HDAC1 (1 peptide), and RbAP46 (1 peptide). All those proteins are the components of the Mi-2/NuRD complex that plays a key role in transcriptional repression through its association with sequence specific DNA-binding protein.

**Figure 4:**
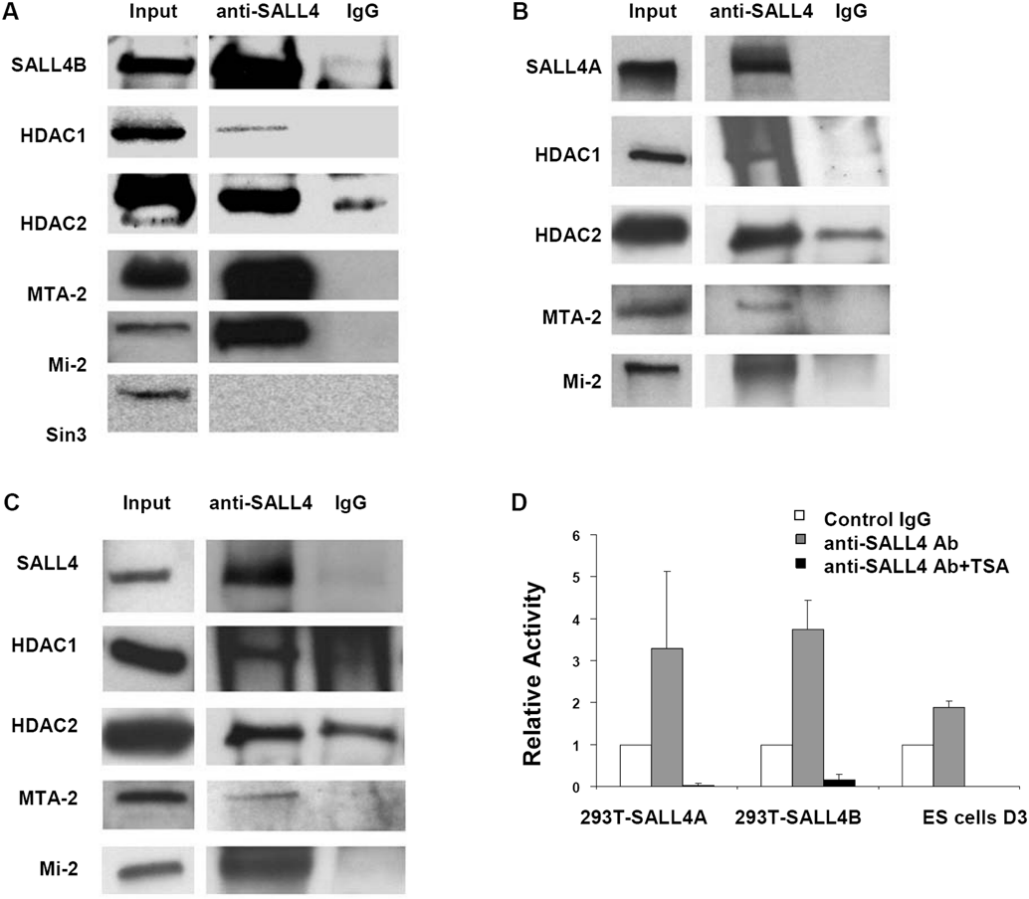
SALL4 associates with Mi-2/NuRD complex and SALL4-interacting protein complex exhibits histone deacetylase (HDAC) activity. Extracts from SALL4B-expressed 293T cells (A), SALL4B-expressed 293T cells (B), or mouse ES cells, (C) were immunoprecipated with an anti-SALL4 antibody or rabbit IgG control followed by Western blotting with indicated antibodies. The HDAC activity of SALL4-bound protein complex was measured using the fluorimetric assay with or without the TSA (D). Data represent at least two independent experiments. Error bars denote standard deviation (SD).

Mass spectrometry results were confirmed by Western blotting analyses (Figure 4A). We observed that HDAC2 (Figure 4A) and RbAP48 (data not shown), the other two components of Mi-2/NuRD complex, were also present as SALL4B-bound proteins. In contrast, Sin3A, a unique protein in the Sin3 histone deacetylase complex, was not detected in SALL4B-containing proteins. This excludes the possibility that SALL4 might be in the Sin3 complex.

To determine whether SALL4A binds to the Mi-2/NuRD complex as well, and whether endogenous SALL4 in general binds to this complex, we did coimmunoprecipitation and Western blotting experiments using the extracts from SALL4A-expressing 293T cells and mouse embryonic stem D3 cells with high endogenous SALL4 expression. It showed that both exogenous SALL4A and endogenous SALL4 were bound to HDAC1, HDAC2, MTA2 and Mi-2 (Figure 4B and 4C). Taken altogether, these results have demonstrated that SALL4 associates with Mi-2/NuRD complex and indicates that SALL4-repressed gene transcription could be mediated by nucleosome remodeling and histone deacetylation.

### SALL4-bound protein complex has histone deacetylase (HDAC) activity

To test whether SALL4-containing protein complex has the capacity to deacetylate histones, we measured its HDAC activity. As shown in Figure 4D, both exogenous SALL4A and SALL4B-containing protein complexes exhibited 2 to 5 fold higher HDAC activity than the IgG control pull-down samples. Similarly, a 2-fold increase in HDAC activity was observed in endogenous SALL4- associated protein complex in ES cells in comparison to the control. Moreover, when both exogenous and endogenous SALL4-interacting protein complexes were incubated with trichostatin A (TSA), a histone deacetylase specific inhibitor, the HDAC activity was dramatically reduced. It suggests that a SALL4-bound protein complex including NuRD components exhibits histone deacetylase activity.

### SALL4 binds to the promoter regions of PTEN and SALL1 and represses their expression *in vitro*

**Figure 5:**
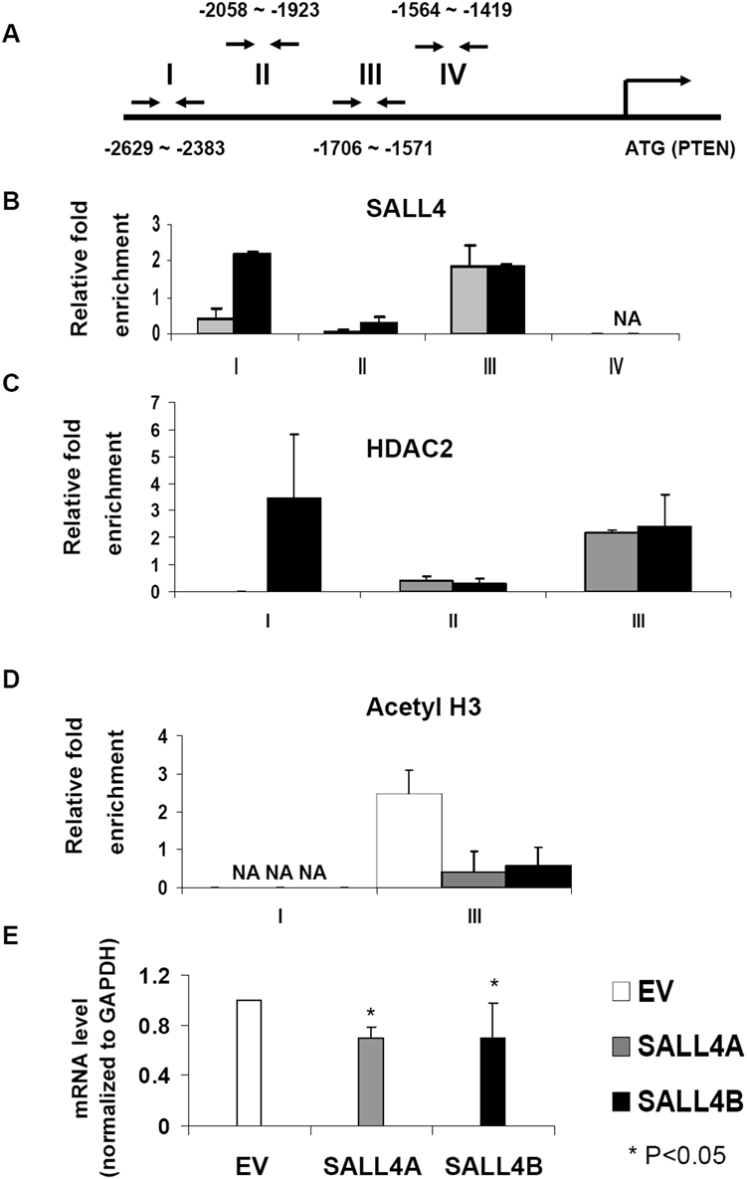
SALL4 co-occupies the same promoter regions of PTEN as HDAC2 and represses its expression *in vitro*. (A) Diagram of PTEN promoter. Antibody against SALL4 was used to immunoprecipitate DNA fragments from SALL4A and SALL4B-expressed 293T cells and fold enrichment was compared to input after normalization with GAPDH. Four pairs of primers in the PTEN promoter region (−2629 bp to −1419 bp) were used in ChIP-qPCR analysis with the ATG site defined as 0. Region I (−2629 bp to −2383 bp) was found to be bound by SALL4B and region III (−1706 bp to −1571 bp) was found to be bound by both SALL4A and SALL4B (B). The same ChIP-qPCR assay was done with an anti-HDAC2 antibody (C) or an anti-acetyl H3 antibody (D). Results are representative of at least two independent experiments. Error bars indicate standard deviations. NA: no amplicon in qPCR. (E) The expression level of PTEN was reduced 30% in SALL4-expressed 293T cells (SALL4A and SALL4B) compared to empty vector controls (EV). The mRNA level was normalized with internal control GAPDH. Data represent as mean±SD, n≥3, P<0.05.

**Figure 6:**
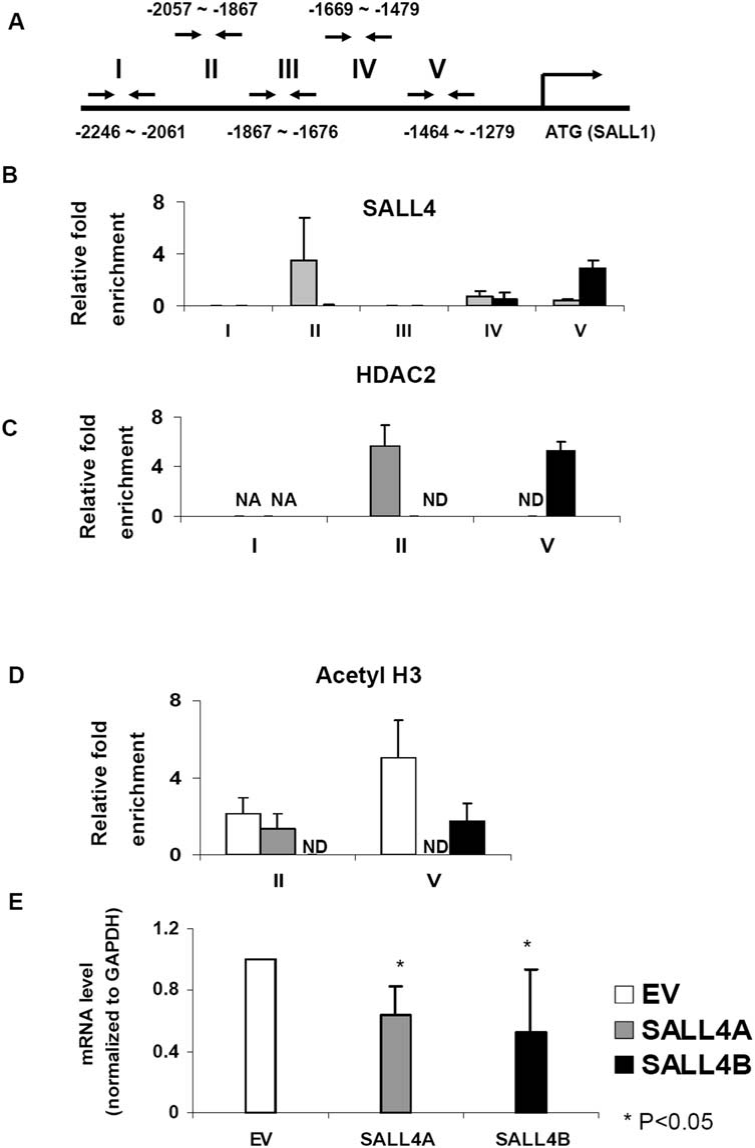
SALL4 co-occupies the same promoter regions of SALL1 as HDAC2 and inhibits its transcription *in vitro*. (A) Diagram of SALL1 promoter. Antibody against SALL4 was used to immunoprecipitate DNA fragments from SALL4A and SALL4B-expressed 293T cells and fold enrichment was compared to input after normalization with GAPDH. Five pairs of primers spanning SALL1 promoter region (−2246 bp to −1279 bp) were used in ChIP-qPCR analysis. Region II (−2057 bp to −1867 bp) was found to be bound by SALL4A and region V (−1464 bp to −1279 bp) was found to be bound by SALL4B (B). The same ChIP-qPCR assay was done with an anti-HDAC2 antibody (C) or an anti-acetyl H3 antibody (D). Results are representative of at least two independent experiments. Error bars indicate standard deviations. NA: no amplicon in qPCR. ND: not done. (E) The mRNA level of SALL1 was reduced 35% and 45% in SALL4A and SALL4B-expressed 293T cells (SALL4A and SALL4B) compared to empty vector controls (EV). The expression level was normalized with internal control GAPDH. Data represent as mean±SD, n≥3, P<0.05.

To determine the molecular mechanism(s) of SALL4 in normal hematopoiesis, leukemogenesis and ESC development, we have mapped SALL4 global gene targets using chromatin-immunoprecipitation followed by microarray hybridization (ChIP-on-chip) in normal human CD34^+^ bone marrow cells, myeloid leukemic NB4 cells and human ESCs. Two genes that have been implicated in ESC, kidney development and leukemogenesis, PTEN and SALL1, were identified as potential SALL4 downstream target genes. We then sought to validate whether SALL4 binds to the promoter regions of these two genes using regular ChIP coupled with qPCR. Chromatin from 293T cells expressing either SALL4A or SALL4B was prepared, sonicated and immunoprecipitated with a rabbit polyclonal antibody against SALL4. QPCR was carried out with a series of primers specific for PTEN and SALL1 promoters, which were designed based on ChIP-on-chip results and covered possible SALL4 binding sites and adjacent non-binding sequences (Figure 5A & 6A). We observed a 2–3 fold enrichment in SALL4 pull-down at its binding site in the PTEN and SALL1 promoters when compared to input (no antibody added), while there was no enrichment at the non-binding sites (Figure 5B and 6B). We also observed that SALL4 was able to bind the promoter regions of these two genes in either mouse ESCs, two human AML samples including M0 (FAB classification) or AML transformed from CML (chronic myeloid leukemia) using ChIP-on-ChIP assays (data not shown).

Interestingly, SALL4A and SALL4B exhibited different binding sites at the promoter regions of PTEN and SALL1. This could be due to the differences between the two isoforms in DNA binding zinc finger domains. While both SALL4A and SALL4B share the same N- and C-terminal domains, SALL4A has eight zinc finger domains, and SALL4B has only three. This structural variation may result in different binding affinity of SALL4A and SALL4B to the DNA and may explain why their binding sites at the promoters of PTEN and SALL1 are not identical.

Moreover, with the aim of determining the regulation of PTEN and SALL1 by SALL4, we examined the expression level of PTEN and SALL1 in 293T cells overexpressing SALL4 using qRT-PCR. We observed that compared to cells transfected with the empty vector, the expression level of PTEN and SALL1 in SALL4A-expressed cells was decreased by 30% and 35%, respectively. Similarly, when SALL4B was overexpressed, the mRNA level of PTEN or SALL1 was reduced by 30% and 50% respectively (Figure 5E & 6E). Altogether, these results demonstrated that both PTEN and SALL1 are direct down-stream target genes of SALL4 and their expressions are negatively regulated by SALL4.

### SALL4 and Mi-2/NuRD complex co-occupy the same genomic binding sites at the promoter regions of PTEN and SALL1

As shown above, SALL4 associates with the Mi-2/NuRD complex that is known to be involved in transcriptional repression. Thus, we propose that SALL4 represses the expression of PTEN and SALL1 via the NuRD complex. To test this hypothesis, we performed ChIP-qPCR analysis using the anti-HDAC2 antibody and examined whether HDAC2 and SALL4 shared the same binding site(s) at the promoters of PTEN and SALL1. We have shown that SALL4 binds to the specific promoter regions of PTEN (−2629 bp∼−2383 bp, Region I, and −1706 bp∼−1571 bp, Region III) and SALL1 (−2057 bp∼−1867 bp, Region II, and −1464 bp∼−1279 bp, Region V). In this experiment, in HDAC2 pulldown fragments of both SALL4A and SALL4B transfected cells, 2 to 5 fold enrichment was detected at the SALL4 binding sites of PTEN and SALL1 promoters when compared with input. However, there was no enrichment observed in the non-binding sites of both genes (Figure 5C & 6C). The same result was obtained from the ChIP-qPCR assay using anti-MTA2 antibody (data not shown). Therefore, these results illustrate that SALL4 and NuRD complex co-occupy the same promoter sites of PTEN and SALL1 and indicate that SALL4 downregulates the transcription of those two genes through histone deacetylation. In addition, we performed the same analysis using anti-acetyl Histone H3 antibody. This analysis showed when SALL4 was overexpressed the enrichment at SALL4-binding sites of PTEN and SALL1 was decreased from 35% to 83% respectively (Figure 5D & 6D), which suggested that there was reduced acetylation of histones H3 at these binding regions. These results strengthen the notion that SALL4-NuRD protein complex suppresses the expressions of PTEN and SALL1, the two SALL4 direct downstream target genes, by deacetylating the histones at their promoter regions.

### SALL4 inhibits expression of PTEN and SALL1 *in vivo*

**Figure 7:**
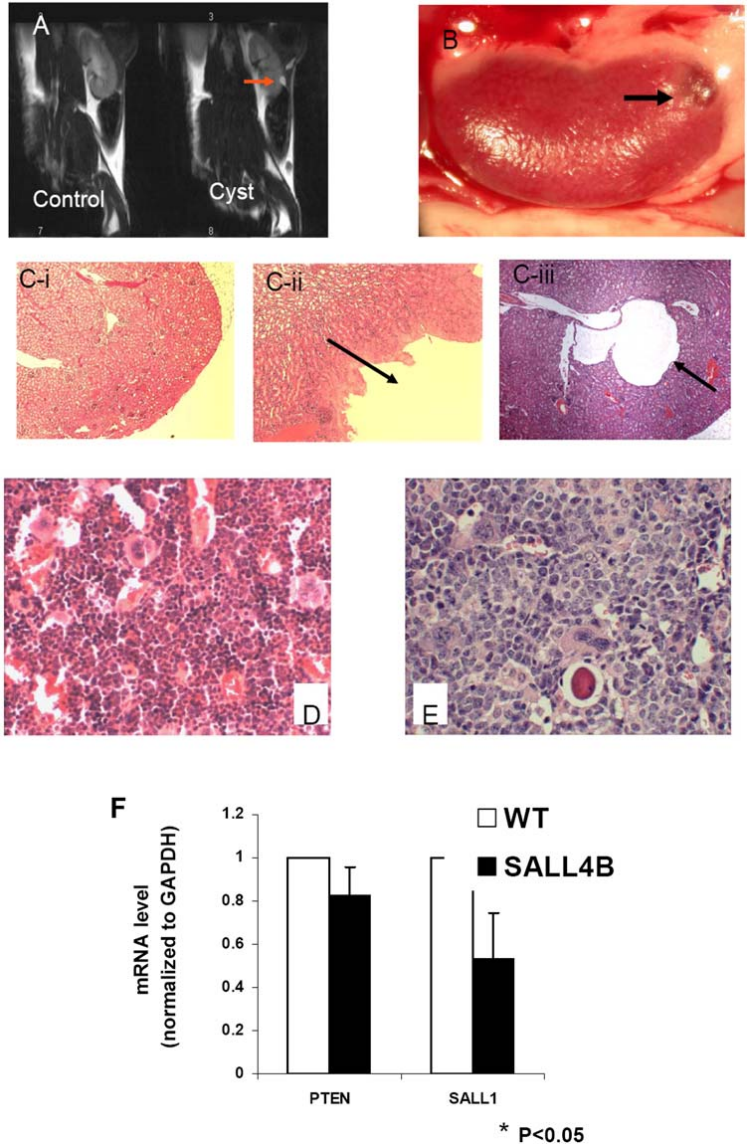
SALL4 represses the transcriptions of PTEN and SALL1 *in vivo*. Cystic kidneys were observed in 50% of the SALL4B transgenic mice. (A) MRI imaging on mouse kidneys. Compared to the control normal kidney, the cystic kidney had a 1.35 mm cyst that is indicated by an arrow. The gross picture of a cystic kidney was shown in (B) with the arrow indicating the cystic area. (C) Histological morphologies on normal kidney (C–I, magnification: ×40) and cystic kidneys (C-ii and C-iii indicated by the arrow, ×40) were presented after stained with Hematoxylin and Eosin (H&E stain). (D) Normal bone marrow from control mice (H&E stain with magnification: ×100). (E) Leukemic bone marrow from transgenic mice (H&E stain with magnification: ×100). (F) The transcriptions of PTEN or SALL1 were decreased 20% and 45% respectively in SALL4B transgenic mice BM or kidney samples. Data represent as mean±SD, n = 4, P<0.05.

We have generated a SALL4B transgenic mice mouse model. We observed that 50% of examined SALL4B transgenic mice had cystic kidneys (Table 1 and Figure 7A–C), which suggested that SALL4 could be involved in kidney development. Since SALL1 is considered as one of the key factors in kidney development, we checked the expression of SALL1 in the kidneys of these transgenic mice. Consistent with *in vitro* studies, the transcription of SALL1 in transgenic mouse kidneys was decreased to 45% of those in wild type controls (Figure 7F).

**Table 1:** Phenotypical characterization of SALL4B transgenic mice.

ID number	Founder	Gender	Age (month)	Phenotype
448	448	f	19	AML
464	464	m	19	MDS
4	464	m	22	Cystic kidney/MDS
88	504	m	2	Cystic kidney/MDS
89	504	m	4	Cystic kidney/MDS
90	504	m	8	AML
86	504	f	18	AML
87	504	f	8	AML
2234	504	m	3	Cystic kidney/MDS
1879	504	m	4	MDS
2837	504	m	4	Cystic kidney/MDS
504	504	m	19	Cystic kidney and MDS
1959	506	m	1	Cystic Kidney
506	506	m	19	Cystic kidney and MDS
25	507	m	8	Cystic Kidney/AML
26	507	m	14	Cystic kidney/MDS
27	507	m	22	MDS/AML
507	507	f	24	AML

SALL4B transgenic mice also displayed MDS-like symptoms and subsequent AML transformation ^20^ (Figure 7 D&E). Since PTEN, a target of SALL4, plays an essential role in hematopoietic stem cell self-renewal and leukemogenesis, we further explored whether regulation of PTEN expression by SALL4 was involved in SALL4 induced AML *in vivo*. The mRNA level of PTEN in the bone marrow (BM) samples obtained from SALL4B transgenic mice was examined by qRT-PCR. It was observed that the expression level of PTEN was decreased 20% in BM cells of SALL4B transgenic mice compared to litter mate controls (Figure 7F).

These findings indicated that the association of SALL4 and Mi-2/NuRD complex could be related to multiple biological processes, and the inhibition of PTEN and SALL1 expression by SALL4 may at least partially contribute to SALL4 induced AML and cystic kidneys in the transgenic mice.

## Discussion

Zinc-finger transcription factor SALL4 is essential for human embryonic development. It plays an essential role in the maintenance of pluripotency and self-renewal properties of embryonic stem cells (ESC) by activation of another pluripotency factor, Oct4. In addition, we have shown that constitutive expression of SALL4 contributes to leukemogenesis in adult mice by activating Bmi-1 [42], a key regulator in hematopoietic stem cells (HSCs) and leukemic stem cells (LSCs) [43]. Parallel loss-of-function studies have also demonstrated that SALL4 is essential for the survival of leukemic cells, and the apoptotic phenotype that is caused by down-regulation of SALL4 can be rescued by overexpression of Bmi-1 [44]. It appears that SALL4 is a unique gene that is involved in self-renewal in ESC, HSC and LSC, in part by activation of stem cell factors. To our best knowledge, SALL4 may be one of the few genes that create such a connection. Thus, further studies on the mechanism(s) of SALL4 function will add value and advance the field of stem cell research.

We have mapped SALL4 global gene targets using chromatin-immunoprecipitation followed by microarray hybridization (ChIP-on-chip) in three types of cells that are related to SALL4 biological functions: leukemic cells [42], ESCs, and primary normal CD34+ cells (unpublished data), which have been correlated with the expression of these gene targets. During these studies we have noticed that SALL4 activates as well as represses its target genes. In addition, SALL1, another SALL gene family member, is capable of repressing transcriptional activity. These findings prompted us to ask whether SALL4 had transcriptional repression activity.

**Figure 8:**
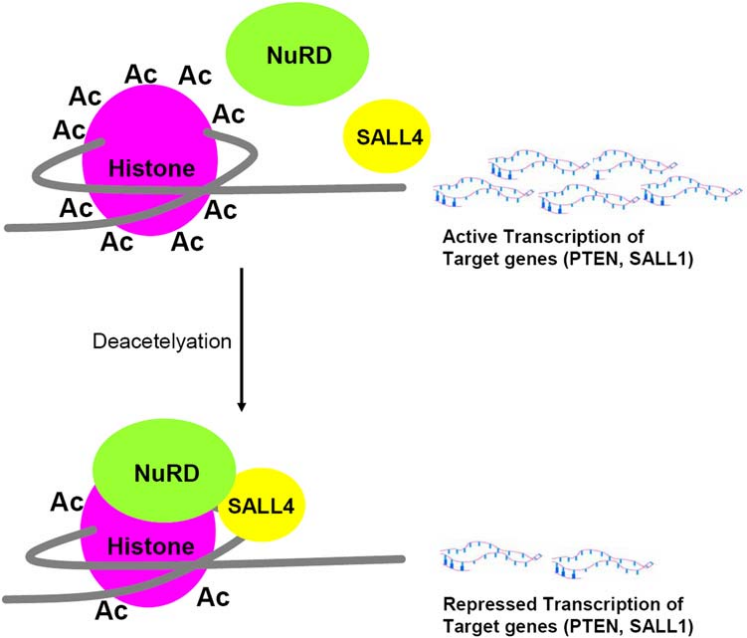
Proposed functional model for SALL4/NuRD complex. SALL4 recruits the NuRD complex to the specific promoter regions on its target genes (i.e. PTEN and SALL1). This results in histone deacetylation at these regions, which leads to condensed heterochromatin and repressed gene expressions.

In this study, we have shown that SALL4 can act as a repressor in some context. More importantly, we have made a novel observation that SALL4 directly connects the epigenetic modulators (NuRD repressor complex) to its downstream target genes, such as PTEN and SALL1 (Figure 8). The NuRD complex has both histone deacetylase and ATP-dependent nucleosome remodeling activity. It plays roles in transcriptional repression through its association with sequence specific DNA-binding protein in ESC, development, hematopoietic differentiation and tumorigenesis [25], [26], [45]–[47]. We have shown through tandem mass spectrometry and/or coimmunoprecipitation that both the endogenous and the exogenous SALL4 can bind to the NuRD complex. In addition, the SALL4-bound protein complex has histone deacetylase (HDAC) activity. To make our study relevant to the biological functions of SALL4, we have searched genes that are involved in ESC, leukemogenesis, and kidney development, and have chosen PTEN and SALL1 as exemplar target genes to demonstrate the mechanism of SALL4-mediated gene repression. Both SALL4 isoforms, SALL4A and SALL4B, can interact with this complex to regulate their down stream targets (PTEN and SALL1), although they do so through different binding sites on the promoter regions of these genes. The SALL4-mediated repression of the expression of PTEN and SALL1 has been confirmed both *in vitro* and *in vivo* and is associated with phenotypes observed in SALL4B transgenic mice, i.e. AML and cystic kidneys, respectively. While both genes are involved in ESCs, kidney development and leukemogenesis, SALL1 is best studied for its essential role in kidney development, and PTEN is known as a key regulator in leukemic stem cells. Therefore, we tested the expression levels of these two genes in their most functionally relevant tissues: SALL1 in kidneys and PTEN in the bone marrow. Both genes were down-regulated in the SALL4B transgenic mice in comparison to their wild type littermates. In addition, our previous study on downregulation of SALL4 in leukemic NB4 cells has demonstrated that PTEN is upregulaetd when the expression of SALL4 is knocking down [42].

**Figure 9:**
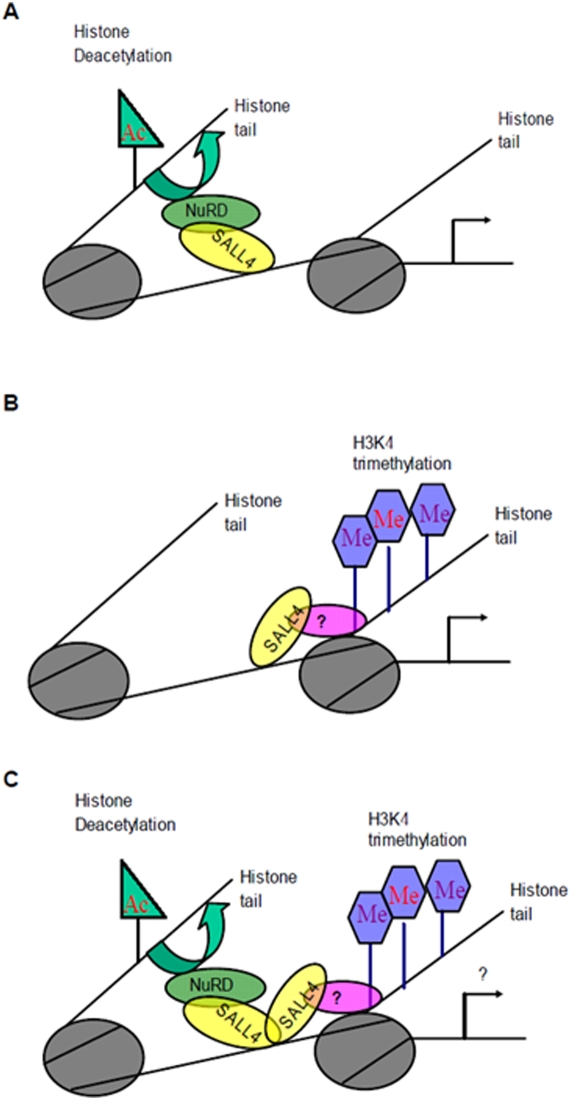
Working hypothesis:SALL4 recruits epigenetic modulators to specific DNA sequences on the promoter regions of its downstream targets. A, SALL4 represses its downstream targets by recruiting NuRD to specific promoter regions that results in histone deacetylation and transcription repression. B, SALL4 activates its downstream targets by recruiting a histone methyltransferase complex to specific promoter regions that results in H3-K4 trimethylation and transcription activation. C, Alternatively, SALL4 recruits both complexes to specific regions on the promoters of target genes. It can either repress or activate the transcription of these genes by balancing the interactions with both complexes.

Our early study on SALL4-mediated activation of Bmi-1 suggests that SALL4 may be involved in epigenetic modification(s), such as trimethylation of histone 3 lysine 4 (H3K4), which is associated with gene activation [48]. Additional study in our lab has indicated that the SALL4-bound protein complex has histone H3K4 trimethylation activity (unpublished data). In this study, we have discovered that SALL4 can interact with an epigenetic repressor complex, NuRD, in gene silencing (Figure 8). Taken together, we have made the novel observation that SALL4 connects the epigenetic modulators (both the activator and repressor complex) to its downstream target genes, a phenomenon that has not been reported for other stem cell proteins, such as Oct4, Nanog, and Sox2. Though further studies are needed to define the precise mechanism(s) that determine SALL4 to work as an activator versus a repressor, this observation should provide us with a unique opportunity to study the connection between the genetic and epigenetic programs in stem cells in the future. Furthermore, we propose the following working model: it is possible that SALL4 recruits epigenetic modulators to specific DNA sequences in the promoter regions of its downstream targets for either activation or repression (Figure 9).

The genetic program in ESC consists of a core transcriptional network including SALL4, OCT4, SOX2, and NANOG. The epigenetic state of an ESC is very unique and is described as “bivalent”, wherein markers for repression (trimethylation of histone 3 lysine 27, H3-K27) and activation (trimethylation of histone 3 lysine 4, H3-K4) on certain developmental genes coexist. This unique epigenetic state is essential for ESC pluripotency [49], [50]. It remains unknown how the core transcriptional network is linked to this unique ‘stem cell” epigenetic state in maintaining properties of ESCs. Our studies suggest that SALL4 may be the key element that connects the epigenetic and genetic programs in ESCs. It is possible that SALL4 recognizes specific DNA binding sites for epigenetic modulators and activates or represses a SALL4 dependent transcriptional network to regulate the self-renewal and pluripotency in ESCs.

Being a pluripotency gene, SALL4 is present at a very early stage along with Oct4 during development, earlier than Nanog. Unlike Oct4 and Nanog, it is present in adult tissue stem cells (such as hematopoietic stem cells) and cancer stem cells (such as leukemic stem cells). The unique role of SALL4 in the self-renewal properties shared by ESCs, HSCs, and LSCs could be associated with its ability to recruit epigenetic modulators to regulate various downstream targets in various stem cells. Our study on SALL4 as a repressor and its association with the NuRD complex is only the first step in understanding the SALL4-mediated epigenetic modification in ESC, adult stem cells and cancer stem cells. Future studies on SALL4 in various stem cell models should help us to address the fundamental connection between chromatin structure and cell function.
