# Correction: Stem Cell Factor SALL4 Represses the Transcriptions of PTEN and SALL1 through an Epigenetic Repressor Complex

**DOI:** 10.1371/annotation/e0bd3a57-1ce4-4eaf-83de-034d39cac787

**Published:** 2009-07-27

**Authors:** Jiayun Lu, Ha-Won Jeong, Nikki Kong, Youyang Yang, John Carroll, Hongbo R. Luo, Leslie E. Silberstein, YupoMa, Li Chai

The second author's name is spelled incorrectly. The correct spelling is Ha-Won Jeong. The co-contribution is still valid. 

